# Exposure to Kynurenic Acid during Adolescence Increases Sign-Tracking and Impairs Long-Term Potentiation in Adulthood

**DOI:** 10.3389/fnbeh.2014.00451

**Published:** 2015-01-06

**Authors:** Nicole E. DeAngeli, Travis P. Todd, Stephen E. Chang, Hermes H. Yeh, Pamela W. Yeh, David J. Bucci

**Affiliations:** ^1^Department of Psychological and Brain Sciences, Dartmouth College, Hanover, NH, USA; ^2^Department of Physiology and Neurobiology, Geisel School of Medicine, Dartmouth College, Hanover, NH, USA

**Keywords:** autoshaping, schizophrenia, NMDA, nicotinic, kynurenine, addiction, incentive salience, motivation

## Abstract

Changes in brain reward systems are thought to contribute significantly to the cognitive and behavioral impairments of schizophrenia, as well as the propensity to develop co-occurring substance abuse disorders. Presently, there are few treatments for persons with a dual diagnosis and little is known about the neural substrates that underlie co-occurring schizophrenia and substance abuse. One goal of the present study was to determine if a change in the concentration of kynurenic acid (KYNA), a tryptophan metabolite that is increased in the brains of people with schizophrenia, affects reward-related behavior. KYNA is an endogenous antagonist of NMDA glutamate receptors and α7 nicotinic acetylcholine receptors, both of which are critically involved in neurodevelopment, plasticity, and behavior. In Experiment 1, rats were treated throughout adolescence with L-kynurenine (L-KYN), the precursor of KYNA. As adults, the rats were tested drug-free in an autoshaping procedure in which a lever was paired with food. Rats treated with L-KYN during adolescence exhibited increased sign-tracking behavior (lever pressing) when they were tested as adults. Sign-tracking is thought to reflect the lever acquiring incentive salience (motivational value) as a result of its pairing with reward. Thus, KYNA exposure may increase the incentive salience of cues associated with reward, perhaps contributing to an increase in sensitivity to drug-related cues in persons with schizophrenia. In Experiment 2, we tested the effects of exposure to KYNA during adolescence on hippocampal long-term potentiation (LTP). Rats treated with L-KYN exhibited no LTP after a burst of high-frequency stimulation that was sufficient to produce robust LTP in vehicle-treated rats. This finding represents the first demonstrated consequence of elevated KYNA concentration during development and provides insight into the basis for cognitive and behavioral deficits that result from exposure to KYNA during adolescence.

## Introduction

Kynurenic acid (KYNA) is a final product of tryptophan metabolism that is synthesized and released in the central nervous system by astroglia (Schwarcz and Pellicciari, 2002). The concentrations of KYNA and its precursor, kynurenine, are significantly increased in the brains of persons with schizophrenia (Erhardt et al., 2001; Schwarcz et al., 2001; Linderholm et al., 2012) and growing evidence indicates that KYNA may contribute to the cognitive deficits that are associated with the disorder. Indeed, KYNA is an endogenous antagonist of NMDA glutamate receptors (NMDA-Rs) and α7 nicotinic acetylcholine receptors (α7-nAChRs; Hilmas et al., 2001; Parsons et al., 1997; Pereira et al., 2002; Stone, 1993), thus an increase in KYNA concentration is likely to impair functions that depend on these receptors, such as attention, learning, and memory (Bast et al., 2003; Gould and Higgins, 2003; Bloem et al., 2014). Consistent with this, a growing number of studies provide evidence of a causal role for KYNA in cognitive dysfunction by showing that an acute increase in KYNA in adult rats impairs cognitive domains that are affected in schizophrenia, including attention (Chess and Bucci, 2006; Alexander et al., 2012), sensory gating (Shepard et al., 2003; Erhardt et al., 2004), context memory (Chess et al., 2009), and spatial memory (Chess et al., 2007).

More recent studies have focused on the effects of KYNA exposure earlier in life, since KYNA levels are likely increased for much longer periods of time in persons with schizophrenia and become elevated earlier than adulthood (Miller et al., 2004, 2006, 2008; Holtze et al., 2008; Asp et al., 2010). This has important ramifications because NMDA-Rs and α7-nAChRs are also essential for neural plasticity and brain development (Komuro and Rakic, 1993; Broide and Leslie, 1999), thus exposure to high levels of KYNA early in life may lead to lasting cognitive and behavioral impairments later in adulthood. In line with this, it has been shown that an increase in KYNA throughout adolescence impaired contextual memory (Akagbosu et al., 2012) and social behavior (Trecartin and Bucci, 2011) when rats were subsequently tested drug free in adulthood. Similarly, treating rat dams with food enriched with l-kynurenine (L-KYN) from gestational day 15 to postnatal day (PND) 21 increased KYNA concentration in the offspring and impaired cognition (Pocivavsek et al., 2012; Alexander et al., 2013).

The present study extended this work in two important directions. Schizophrenia is associated with dysfunctional brain reward circuitry, particularly the midbrain dopamine system. Patients have higher levels of dopamine synthesis and release compared to age-matched controls (Carlsson et al., 2001) and elevated levels of KYNA may contribute to this abnormal pattern of dopamine activity. In rodents, pharmacologically elevated levels of endogenous KYNA increase the firing rate and bursting activity of dopamine neurons in the ventral tegmental area (VTA), an effect mediated by NMDA-Rs (Erhardt and Engberg, 2002). In addition, manipulating KYNA levels with cyclooxygenase inhibitors similarly alters the activity of VTA dopamine neurons (Schwieler et al., 2006). Furthermore, elevation of KYNA levels increases dopamine release in the nucleus accumbens (NAc; Nilsson-Todd et al., 2007), a structure that is involved in reward learning and addiction (e.g., Robinson and Berridge, 1993; Di Chiara, 2002; Kelley, 2004). Thus, Experiment 1 tested whether an increase in KYNA concentration during adolescence impacts reward-related behavior and motivation. Rats were treated with 100 mg/kg of L-KYN throughout adolescence, which results in a three- to fourfold increase in KYNA levels (Erhardt et al., 2004; Akagbosu et al., 2012) and is consistent with the increases observed in persons with schizophrenia (Erhardt et al., 2001; Schwarcz et al., 2001). As adults, the rats were tested drug-free in an autoshaping procedure in which one lever conditioned stimulus (CS) was presented for a short period of time and followed immediately by delivery of a food unconditioned stimulus (US) upon lever retraction, while a second lever was not paired with food (Chang et al., 2012). Although food delivery is not contingent on the rat’s behavior, rats will typically approach, contact, and bite the lever CS that is paired with the US (Davey and Cleland, 1982). These CS-directed behaviors (also known as sign-tracking, e.g., Flagel et al., 2011) are thought to reflect the lever acquiring incentive salience (motivational value) as a result of its pairing with the US (e.g., Berridge, 2004). Importantly, sign-tracking has been shown to be mediated by the NAc and its dopaminergic inputs from the VTA (Flagel et al., 2011; Chang et al., 2012; Saunders and Robinson, 2012). Substance abuse and addiction are associated with increased sensitivity to drug-related cues, perhaps resulting from sensitization of midbrain dopamine neurons in response to drugs that imbues the drug-related stimuli with excessive incentive salience (Robinson and Berridge, 1993, 2001). Since KYNA enhances dopaminergic neurotransmission in midbrain reward circuits (Erhardt and Engberg, 2002), we predicted that rats exposed to KYNA during adolescence would exhibit excessive sign-tracking (lever pressing) as adults. Importantly, behavioral testing took place beginning on PND 100, which is well into adulthood and when KYNA levels are no longer elevated following this treatment regimen (Akagbosu et al., 2012). Thus, any behavioral differences observed between L-KYN-treated rats and vehicle-treated rats could not be attributed to differences in KYNA concentration at the time of testing.

The second goal of the current study was to determine how exposure to KYNA during adolescence alters neural function in adulthood. Indeed, despite the demonstration that cognitive deficits result from increasing KYNA levels during adolescence (Trecartin and Bucci, 2011; Akagbosu et al., 2012), no study has investigated the neural substrates underlying those deficits. Thus, we examined the effects of L-KYN treatment during adolescence on the ability of adult hippocampal neurons to undergo long-term potentiation (LTP). We chose to study hippocampal LTP because of the recent interest in hippocampal involvement in autoshaping and because it has been shown previously that exposure to KYNA during adolescence impairs hippocampal-dependent behavior (Akagbosu et al., 2012). We predicted that LTP would be reduced in rats treated with L-KYN during adolescence compared to control rats.

## Materials and Methods

### Experiment 1

#### Subjects

Sixteen male Long–Evans rats were obtained from Harlan Laboratories (Indianapolis, IN, USA) at 21 days of age. Rats were housed in groups of 4 upon arrival with food and tap water available *ad libitum* (Purina standard rat chow; Nestle Purina). Rats were allowed 6 days to acclimate to the vivarium before drug treatment began. When they reached 63 days old, the rats were separated into individual cages and gradually food restricted to 85% of their baseline free-feeding body weights over the next 7 days. Weights were measured daily and maintained by supplementing with rat chow. Rats were maintained on a 14:10 light-dark schedule (lights on at 7:00 a.m., off at 9:00 p.m.) throughout the study and monitored and cared for in compliance with the Association for Assessment and Accreditation of Laboratory Animal Care guidelines and the Dartmouth College Institutional Animal Care and Use Committee.

#### Drug preparation

L-KYN (SAI Chemicals, India) was prepared fresh daily by dissolving in 2N sodium hydroxide and adding 0.1M 4-(2-hydroxyethyl)-1-piperazineethanesulfonic acid (HEPES) buffer to bring it to a final concentration of 30 mg/ml. The solution was then brought to a neutral pH by adding drops of 1N hydrochloric acid.

#### Treatment regimen

On PND 27, rats were quasi-randomly assigned to either the L-KYN-treated group or the vehicle-treated control group (*n* = 8/group) with two of each set of four group-housed rats assigned to each condition. On PND 27-29, each rat received a daily intraperitoneal injection of either the L-KYN solution (100 mg/kg) or a comparable volume of 0.1M HEPES buffer (vehicle). Injections were administered on alternate sides of the abdomen to reduce discomfort at the injection site. On PND 30–32, no injections were administered and the rats were not handled. This 3-day-on/3-day-off drug treatment regimen was repeated another 4 times resulting in a total of 15 injections of L-KYN (or vehicle), the last of which occurred on PND 53. As shown previously, this procedure increases KYNA concentration fourfold on days when rats are treated with L-KYN (Akagbosu et al., 2012). Compared to injecting rats every day from PND 27–53, the 3-day injection cycle reduces distress and sensitivity at the injection site and also minimizes the potential for metabolic adaptations following chronic systemic L-KYN treatment for more than 3 consecutive days (Vecsei et al., 1992).

#### Behavioral apparatus

The behavioral procedures were carried out in eight standard conditioning chambers (Med Associates, Georgia, VT, USA) enclosed in sound-attenuating cubicles (62 × 56 × 56 cm) outfitted with exhaust fans to provide airflow and background noise (~68 dB). The chambers consisted of aluminum front and back walls, clear acrylic sides and top, and grid floors composed of stainless steel rods (5 mm diameter) spaced 1.5 cm apart (center to center). Each chamber was outfitted with a food cup that was recessed in the center of the front wall. Retractable levers were positioned to the left and right of the food cup. The levers were 4.8 cm long, positioned 6.2 cm above the grid floor, and protruded 1.9 cm when extended. A 2.8-W house light was mounted 15 cm above the grid floor on the back wall of the chamber and provided background illumination. Four panel lights were also present in the chamber but were not used in this experiment. Three of the lights were located 10.8 cm above the floor, two of them were positioned 6.4 cm to the left or right of the food cup, and one was located directly above the food cup. A fourth panel light was located 15 cm above the floor. The reinforcer was a 45-mg grain-based rodent food pellet (Bioserv, Flemington, NJ, USA). A pair of photobeam sensors was located across the front of the food cup. The levers and food delivery were controlled by a PC computer that also monitored breaks in the photobeam.

#### Behavioral procedure

During PND 70–100, the rats participated in a prior experiment with different visual, tactile, and olfactory stimuli in the test chamber. In that experiment, rats were trained to press the right lever and received several presentations of an auditory cue as well as six mild footshocks (0.5 mA). Because of this previous lever-pressing experience, the following procedure was used to extinguish lever pressing prior to the start of the present experiment. Rats were exposed to the conditioning chambers for 1 h on each of 4 consecutive days. During these 1-h sessions, both levers were continuously extended but responses had no consequence. By the fourth session, the mean rate of responding on the left lever was 0.46 presses/min for rats in the L-KYN group and 0.39 presses/min for rats in the saline group. The mean rate of responding on the right lever was 0.98 presses/min for the L-KYN group and 1.27 presses/min for the saline group.

On the first day of the present experiment, all rats received one 30-min magazine training session in which food pellets were delivered freely on a random time (RT) 30-s schedule, resulting in approximately 60 pellets being delivered. On each of the next 7 days, all rats received a single 60-min session of training. Each session consisted of 25 CS+ and 25 CS− trials with an average intertrial interval (ITI) of 1 min. The CS+ trials consisted of a 10-s extension of one lever and delivery of two food pellets upon retraction of the lever. The CS− trials consisted of a 10-s extension of the other lever with no delivery of food pellets. Trial order was random with the exception that no more than two trials of the same type were allowed to occur consecutively. Levers were counterbalanced so that for half of the rats within each group the CS+ lever was the right lever, and for the other half of the rats the CS+ lever was the left lever.

#### Data analysis

The rate of lever pressing and the percentage of trials in which at least one lever press occurred were recorded, as well as the number of head entries into the food cup and percent of time spent in the food cup. Data were analyzed using a 2 (Group: Vehicle vs. L-KYN) × 2 (Cue: CS+ vs. CS−) × 7 (Session) analyses of variance.

### Experiment 2

#### Subjects and drug treatment

Six male Long–Evans rats were obtained from Harlan Laboratories on PND 21 and maintained as described in Experiment 1. L-KYN or vehicle was prepared and administered as described in Experiment 1 (*n* = 3 rats/group).

#### Preparation of hippocampal slices

On ~PND 70, rats were decapitated and the brains were quickly removed and immersed in ice-cold artificial cerebral spinal fluid (aCSF) saturated with 95% O_2_ and 5% CO_2_. The aCSF contained (in mM): 125 NaCl, 2.5 KCl, 2.5 CaCl, 2.2 H_2_O, 1.3 MgCl_2_, 1.25 NaH_2_PO_4_, 25 Glucose, and 25 NaHCO_3_. Sagittal slices of hippocampus (350 μm) were cut from each brain (3–5 sections/brain) using a vibroslicer (Electron Microscopy Sciences, Hatfield, PA, USA) and incubated for at least 1 h in a recovery chamber prior to recording. Individual slices were then transferred to a recording chamber, perfused continuously with oxygenated aCSF at a flow rate of 3–4 ml/min, and maintained at a temperature of 32 ± 1°C.

#### Electrophysiological recordings

Excitatory postsynaptic field potentials (fEPSP) were recorded in CA1 stratum radiatum (dendritic region) with a glass microelectrode pulled from 1.5-mm fiber-filled capillary tubing using a Brown-Flaming electrode puller (P97, Sutter Instruments, Novato, CA, USA) and filled with 150 mM NaCl. A platinum/iridium concentric bipolar electrode (FHC Inc., Bowdoinham, ME, USa) was placed at the path of the Schaffer collaterals to evoke fEPSPs. Electrical signals were amplified by an Axopatch-1D amplifier (Axon Instruments, Foster, CA, USA). An Intel Pentium-based computer with pCLAMP version 9.2 (Molecular Devices, Sunnyvale, CA, USA) was used for on-line acquisition and off-line analysis of data. LTP was induced at 25 or 50% of maximal amplitude by high-frequency stimulation (HFS; 2 trains at 10-s intervals with each train consisting of pulses delivered at 100 Hz).

#### Statistical analysis

Baseline fEPSPs were collected each min for the 10-min period prior to the induction of LTP. Normalized values were calculated from the average response from all rats. After induction, the peak fEPSP amplitude was measured with respect to the baseline every minute for a total of 42 min. Mean baseline data and mean post-induction data were analyzed using a repeated measures ANOVA with Group (vehicle, L-KYN) as the between-subjects variable and Time (pre, post) as the within-subjects variable. Interactions were decomposed using pairwise *t*-tests. An alpha level of 0.05 was used for all analyses.

## Results

### Experiment 1

One L-KYN-treated rat was excluded from the data analysis because responding was 3 standard deviations lower than the group mean (a response was made on only 6.9% of the CS+ presentations; the next lowest in the L-KYN group was 83.4%).

#### Lever pressing

##### Response rate

**Figure 1:**
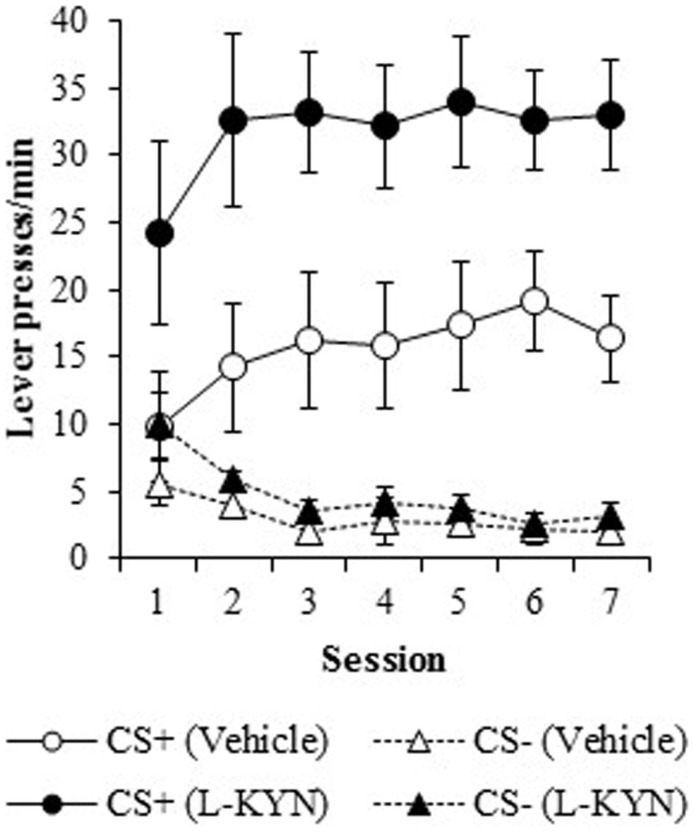
**Lever-pressing behavior (responses/min) during the autoshaping procedure in Experiment 1**. Compared to vehicle-treated rats, those that were treated with L-KYN throughout adolescence exhibited an increase in sign-tracking as evidenced by significantly higher rates of responding when they were tested as adults. Data are means ± SEM.

As shown in Figure 1, the L-KYN-treated rats exhibited higher rates of sign-tracking to the CS+ (as measured by lever presses per minute) than control rats, while no differences were observed in responding to the CS−. A three-way ANOVA revealed a main effect of Group [*F(*1, 13) = 10.4, *p* < 0.01] and a significant main effect of Cue [*F*(1, 13) = 63.5, *p* < 0.001]. The ANOVA also revealed significant interactions between Group and Cue [*F*(1, 13) = 8.5, *p* < 0.02] and Cue and Session [*F*(6, 78) = 4.5, *p* < 0.001]. The main effect of Session was not statistically significant (*p* > 0.9), nor were the Group × Session (*p* > 0.9) or Group × Cue × Session (*p* > 0.9) interactions.

To assess the source of the critical Group × Cue interaction, a two-way ANOVA for the CS+ data revealed a significant main effect of Group [*F*(1, 13) = 9.9, *p* < 0.01]. There was neither significant main effect of Session (*p* > 0.1) nor a significant Group × Session interaction (*p* > 0.9). A two-way ANOVA for the CS− cue revealed a significant main effect of Session [*F*(6, 78) = 7.1, *p* < 0.001], but no main effect of Group (*p* > 0.2) nor a Group × Session interaction (*p* > 0.6). Thus, L-KYN treated rats responded more to the CS+ than vehicle-treated rats, but responding to the CS− was not different between groups.

##### Percentage of trials with a response

**Figure 2:**
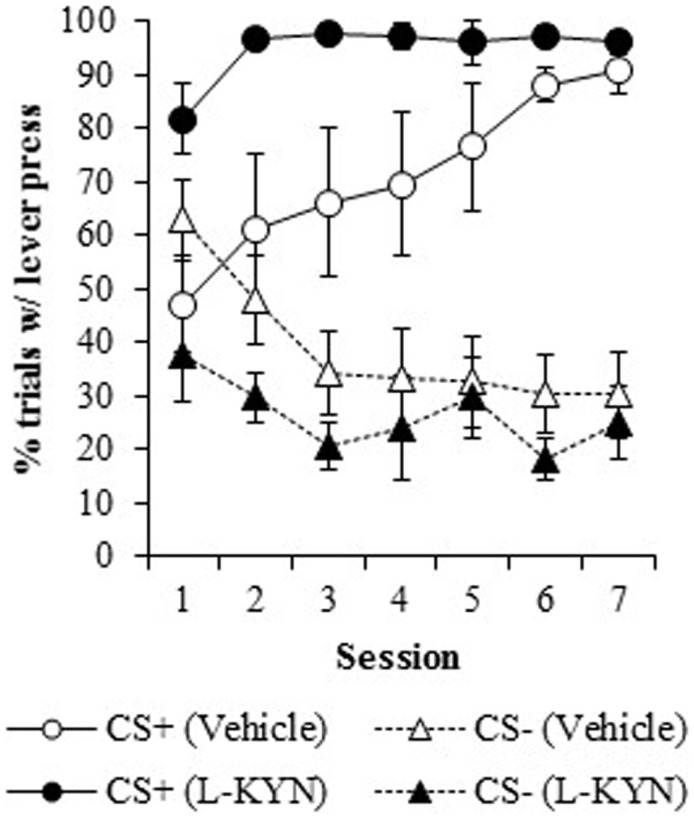
**The percentage of trials in which a lever press occurred in Experiment 1**. Compared to vehicle-treated rats, those that were treated with L-KYN throughout adolescence exhibited an increase in sign-tracking as evidenced by a significantly higher percentage of trials with a lever press when they were tested as adults. Data are means ± SEM.

Figure 2 illustrates the percentage of trials with at least one lever press during the presentation of the CS+ or the CS−. L-KYN-treated rats initially responded on a greater percentage of CS+ trials than the vehicle-treated rats, but the vehicle-treated rats eventually reached a similar level of responding by day 7. A three-way ANOVA revealed a main effect of Group [*F*(1, 13) = 8.3, *p* < 0.02]. There was also a significant effect of Cue [*F*(6, 78) = 59.9, *p* < 0.001] and a significant interaction between Session and Cue [*F*(6, 78) = 10.5, *p* < 0.001], indicating a change in responding to each cue across time. The main effect of Session was not significant (*p* > 0.8) and neither were any of the interactions (Group × Cue, *p* > 0.5; Group × Session, *p* > 0.1; Group × Cue × Session, *p* > 0.6).

A two-way ANOVA used to further investigate the effect of chronic adolescent L-KYN administration on responding to the CS+ revealed a significant main effect of Group [*F*(1, 13) = 6.1, *p* < 0.03] and a significant main effect of Session [*F*(6, 78) = 5.8, *p* < 0.001]. A significant Group × Session interaction was also detected [*F*(6, 78) = 2.3, *p* < 0.05], indicating that the L-KYN-treated rats and vehicle-treated rats responded differently to the CS+ across the seven sessions. One-way ANOVAs for each session revealed significant differences between the vehicle and L-KYN groups on day 1 [*F*(1, 13) = 9.2, *p* < 0.01], day 2 [*F*(1, 13) = 5.5, *p* < 0.03], and day 6 [*F*(1, 13) = 5.6, *p* < 0.03], a marginally significant difference on day 3 [*F*(1, 13) = 4.5, *p* = 0.05], and no significant differences between the groups on day 4 (*p* > 0.1), day 5 (*p* > 0.1), or day 7 (*p* > 0.4). For the CS−, only the main effect of Session was significant [*F*(6, 78) = 7.1, *p* < 0.001], indicating that there were no differences between the groups in responding to the CS−. Thus, L-KYN-treated and vehicle-treated rats differed in CS+ responding early but not late in training.

On both measures (response rate and percentage of trials with a response), there was a significant difference between groups beginning on the first session, as described above. To determine if L-KYN-treatment simply resulted in higher levels of unconditioned lever pressing, we analyzed responding during the first 5-trial block in Session 1. There was no group difference in responding on either measure during the first block of trials (*p*s > 0.1), indicating that levels of unconditioned lever pressing did not differ between groups. Instead, the group difference emerged over the course of training.

#### Food cup behavior

A three-way ANOVA of the number of food cup entries during the CS+ and CS− revealed a significant main effect of Cue [*F*(1, 13) = 6.6, *p* < 0.02] and a significant main effect of Session [*F*(6, 78) = 3.1, *p* < 0.01]. Neither the main effect of Group (*p* > 0.2) nor any interactions (Cue × Group, *p* > 0.1; Session × Group, *p* > 0.8; Session × Cue, *p* > 0.2; Cue × Session × group, *p* > 0.2) were statistically significant. Overall, food cup behavior was lower during the CS+ than the CS−, likely due to sign-tracking to the CS+ since lever pressing and food cup behavior are competing responses. For time spent in the food cup, a three-way ANOVA revealed a main effect of Session [*F*(6, 78) = 4.3, *p* < 0.001] and a significant Group × Session interaction [*F*(6, 78) = 2.4, *p* < 0.03]. Analysis of the interaction indicated that during Session 1, the L-KYN and vehicle groups did not discriminate between CS+ and CS− trials. During Session 7, however, L-KYN-treated rats spent more time in the food cup during CS− trials than CS+ trials, while vehicle-treated rats spent comparable amounts of time in the food cup during CS+ and CS− trials (see Table 1). This was likely due to the increased lever pressing during CS+ trials in L-KYN-treated rats. There were no other significant main effects (Cue, *p* > 0.3; Group, *p* > 0.2) or interactions (Cue × Group, *p* > 0.3; Session × Cue, *p* > 0.6; Session × Cue × group, *p* > 0.2) detected.

**Table 1:** **Food cup behavior**.

	L-KYN		Vehicle	
	CS+	CS−	CS+	CS−
Entries/min: Session 1	20.85 ± 4.68	27.63 ± 3.06	30.99 ± 5.04	33.51 ± 4.14
Session 7	7.44 ± 4.59	23.83 ± 6.58	17.52 ± 5.34	25.29 ± 3.72
% Time in food cup: Session 1	20.01 ± 5.40	28.68 ± 5.46	39.64 ± 8.75	38.91 ± 7.17
Session 7	7.33 ± 3.51	24.84 ± 9.39	26.59 ± 11.91	25.61 ± 7.79


### Experiment 2

**Figure 3:**
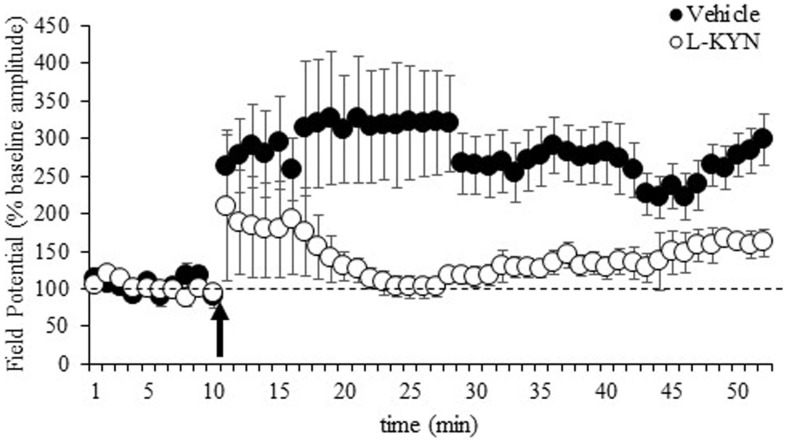
**fEPSPs recorded in Experiment 2 from hippocampal slices from adult rats before and after a burst of high-frequency stimulation (HFS, 100 Hz; arrow)**. Values after HFS are expressed relative to the baseline (average of the responses during the 10-min period prior to LTP induction; dotted line). The burst of HFS induced LTP in vehicle-treated rats but not in rats treated with L-KYN during adolescence. Data are means ± SEM (11 slices from vehicle-treated rats and 15 slices from L-KYN-treated rats).

The LTP data are shown in Figure 3 and represent the average values obtained from 11 sections from vehicle-treated rats and 15 sections from L-KYN-treated rats. A repeated measures ANOVA revealed significant main effects of Group [*F*(1, 24) = 5.7, *p* < 0.03] and Time [*F*(1, 24) = 14.4, *p* < 0.001] and a significant Group × Time interaction [*F*(1, 24) = 5.4, *p* < 0.03]. In the vehicle-treated group, there was a 305 ± 58% increase above baseline in the amplitude of the fEPSP, which was statistically significant [*t*(10) = 3.5, *p* < 0.01]. For L-KYN-treated rats, the post-induction response was only 150 ± 37% above baseline and did not reach statistical significance [*t*(14) = 1.3, *p* > 0.2], indicating that LTP was not induced. In addition, the post-induction response was significantly greater in the controls compared to the L-KYN-treated rats [*t*(24) = 2.6, *p* < 0.03], while there was no group difference in the pre-induction baseline responses [*t*(24) = 1.0, *p* > 0.3].

## Discussion

The present study examined the effects of increased KYNA concentration during adolescence on the brain and behavior in adulthood. Experiment 1 tested the hypothesis that rats treated with L-KYN throughout adolescence, which increases KYNA levels three- to fourfold (Akagbosu et al., 2012), would exhibit increased sensitivity to reward-related cues later in adulthood. Consistent with this hypothesis, we found that L-KYN-treated rats exhibited more sign-tracking behavior compared to vehicle-treated rats when they were tested drug-free as adults. This was manifest as higher rates of lever pressing to the CS+ (a lever paired with food reward) and a greater percentage of trials with a lever press. In contrast, treatment with L-KYN did not increase responding to the CS− (the lever that was not reinforced). These findings indicate that exposure to elevated levels of KYNA during adolescence, a critical time in development, results in an increased sensitivity to reward-related cues later in life.

A similar increase in sensitivity occurs to drug-related cues and is thought to arise from the sensitization of dopamine neurons in response to drugs that imbue the drug-related stimuli with excessive incentive salience (Robinson and Berridge, 1993, 2001). Interestingly, schizophrenia often co-occurs with a variety of substance abuse disorders, particularly alcohol (Green et al., 2008; Koskinen et al., 2009) and nicotine addiction (Lohr and Flynn, 1992; Kumari and Postma, 2005; Brown et al., 2012) and is thought to result from dysfunctional reward circuitry (Green et al., 1999; Chambers et al., 2001). Rats treated subchronically with L-KYN show elevated levels of dopamine release in NAc compared to controls in response to amphetamine injections (Olsson et al., 2009), suggesting that prior treatment with L-KYN may sensitize the midbrain dopamine system and thus enhance the incentive value attributed to rewards and their associated cues. The present finding that exposure to KYNA increases sign-tracking behavior suggests that elevated levels of KYNA in persons with schizophrenia may contribute to the propensity to engage in drug use. Indeed, sign-tracking is mediated by the NAc and its dopaminergic inputs from the VTA (Flagel et al., 2011; Chang et al., 2012; Saunders and Robinson, 2012) and KYNA enhances dopaminergic neurotransmission in midbrain reward circuits (Erhardt and Engberg, 2002). Moreover, dopamine receptor antagonists such as apomorphine, as well as commonly used antipsychotic drugs like clozapine and haloperidol, reduce sign-tracking behavior (Dalley et al., 2002; Danna and Elmer, 2010) while goal-tracking behavior (approaching the reward itself) is unaffected.

In Experiment 2, we found that hippocampal LTP was deficient in adult rats that had been treated with L-KYN during adolescence compared to vehicle-treated controls, indicating that elevated levels of KYNA impair LTP. Conversely, Potter et al. (2010) demonstrated that LTP was enhanced in mice that lacked kynurenine aminotransferase II, the major biosynthetic enzyme of brain KYNA. These mice had significantly lower levels of KYNA compared to wild-type controls. Together with the present findings, this suggests that changes in the concentration of KYNA can bi-directionally modulate the ability of hippocampal neurons to undergo LTP, a process that is critical for normal brain development and cognitive function.

The observed decrease in the ability to undergo LTP is the first identified consequence of exposure to high levels of KYNA during adolescence and provides new insight into the basis of previously reported behavioral deficits. For example, it has been shown that contextual fear conditioning (Akagbosu et al., 2012) and social behavior (Trecartin and Bucci, 2011) are impaired in adult rats that have been exposed to KYNA during adolescence. It is well established that contextual fear memory is dependent on the hippocampus (Maren et al., 1997) and likewise, social behavior is altered by manipulations of hippocampus (Flores et al., 2005). Interestingly, lesions of the hippocampus also affect sign-tracking. Ito et al. (2005) found that hippocampal damage increased sign-tracking behavior, similar to the effects of adolescent L-KYN exposure in the present study. It is important to note, however, that Ito et al. (2005) used a different CS+ and CS− modality (a touch screen) than the one used here, which may involve different brain areas and systems (Chang and Holland, 2013). In contrast, other studies have found that hippocampal damage attenuates sign-tracking (Good and Honey, 1991; Fitzpatrick and Morrow, 2014). In those studies, the lack of a CS− lever may have contributed to the contrasting results. Indeed, it has been shown that different results are sometimes obtained depending on whether a CS− lever is included in the experimental design (Chang et al., 2012). Additionally, only the ventral hippocampus was damaged in the study by Fitzpatrick and Morrow (2014), whereas L-KYN treatment, and the resulting increase in KYNA, likely affected the entire hippocampus. This is important to consider because of the behavioral and anatomical differences between dorsal and ventral subregions of hippocampus (Fanselow and Dong, 2010). Regardless, the present findings suggest that changes in the potential for hippocampal neurons to undergo synaptic plasticity, induced by exposure to elevated levels of KYNA during adolescence, may contribute significantly to the cognitive and behavioral deficits observed later in adulthood. Future studies are needed to expand on this by identifying the changes in hippocampal morphology and/or connectivity that may result from exposure to high levels of KYNA during development. In addition, there are likely to be changes in other brain structures following KYNA exposure and further studies will be needed to determine which behavioral impairments are due to KYNA-induced changes in hippocampus *per se*.

Nonetheless, these findings have several potential implications for understanding the neural substrates of schizophrenia. The cognitive deficits in schizophrenia include similar impairments in hippocampal-dependent processes, including contextual and spatial memory (Cohen et al., 1999; Waters et al., 2004; McClure et al., 2008), sensory gating (Tregellas et al., 2007), and social withdrawal (Sams-Dodd, 1999). Moreover, schizophrenia is thought to involve alterations in hippocampal structure and function (Harrison, 2004), and neonatal ventral hippocampal lesions in rats result in various symptoms of schizophrenia (Tseng et al., 2009; Peleg-Raibstein et al., 2012). KYNA-induced changes in hippocampal function may thus contribute significantly to the cognitive deficits that are often associated with schizophrenia. In addition, it has recently been proposed that the alterations in brain reward systems and dopamine function thought to underlie schizophrenia may be the result of dysfunctional modulatory control by the hippocampus (Grace, 2012). In this way, changes in hippocampal function resulting from high levels of KYNA may lead to dys-regulated reward-related behavior.

The L-KYN injection regimen used here increases KYNA concentration during treatment, but levels return to normal at the time of testing as adults (Akagbosu et al., 2012). Thus, the effects we observed on sign-tracking and on hippocampal LTP cannot be attributed to elevations in KYNA concentration at the time of testing. It is also unlikely that the increase in lever pressing to the CS+ merely reflects a change in baseline activity, since we have previously shown that locomotor activity is unchanged by adolescent exposure to KYNA (Akagbosu et al., 2012). Moreover, there were no group differences in responding to the CS− in the present study. It is possible that the effects of L-KYN administration could be due to changes in the concentration of kynurenine metabolites other than KYNA. However, previous data indicate that a higher dose of L-KYN (150 mg/kg compared to 100 mg/kg in our study) failed to significantly increase levels of quinolinic acid, for example (Shepard et al., 2003). Although the possibility of increasing the concentration of other metabolites could be mitigated by directly injecting synthetic KYNA into the cerebral ventricles, there are several important advantages to elevating KYNA levels by injecting L-KYN. For example, increasing KYNA concentration by administering its precursor allows KYNA to be synthesized only in brain regions that have the neural machinery to produce KYNA. This is important because KYNA acts at receptors that are distributed ubiquitously in the brain, yet the distribution of KYNA-synthesizing astroglia is not uniform and the increase in KYNA concentration in schizophrenia is region-dependent (Schwarcz et al., 2001). In addition, the increase in KYNA observed in schizophrenia is due to upregulation of enzymes such as TDO2, which increases the availability of kynurenine (Miller et al., 2006). Thus, increasing KYNA levels by administering L-KYN lends a high degree of physiological relevance.

Schizophrenia is characterized by a constellation of positive symptoms (e.g., hallucinations), negative symptoms (e.g., social withdrawal), and cognitive deficits (e.g., attention, memory, inhibition). While standard-of-care dopaminergic compounds are often effective in alleviating positive symptoms, the cognitive deficits are notoriously unresponsive to treatment (Blin, 1999) and their cause has remained unclear. Similarly, although the majority of persons diagnosed with schizophrenia also suffer from substance abuse disorders (Green and Brown, 2006; Hartz et al., 2014), few treatment options exist and little is known about the biological link between schizophrenia and substance abuse. The present findings indicate that reward-related behavior and hippocampal synaptic plasticity are compromised in adult rats that had been exposed to high levels of KYNA during adolescence, a critical stage in neural development. Consistent with the notion that KYNA levels may be particularly impactful during development, it has been shown previously that chronic L-KYN treatment during adolescence, but not during adulthood, reduces social behavior (Trecartin and Bucci, 2011). Moreover, the increase in KYNA concentration observed in persons with schizophrenia likely begins early in life (Miller et al., 2004, 2006, 2008; Holtze et al., 2008; Asp et al., 2010), and the symptoms of schizophrenia typically emerge during late adolescence (Harrop and Trower, 2001). However, additional studies are needed to fully differentiate between the behavioral, physiological, and morphological changes induced by exposure to KYNA during adolescence compared to adulthood. Nonetheless, the findings add to a growing literature implicating an elevation in KYNA concentration as a causal factor leading to the cognitive and behavioral symptoms of schizophrenia. The data also support the notion that the development of anti-kynurenergic compounds may provide a new therapeutic avenue for treating schizophrenia (Erhardt et al., 2009; Wonodi and Schwarcz, 2010; Schwarcz et al., 2012; Wu et al., 2014).

## Conflict of Interest Statement

The authors declare that the research was conducted in the absence of any commercial or financial relationships that could be construed as a potential conflict of interest.
